# Medical Colleges

**Published:** 1872-09

**Authors:** 


### MEDICAL COLLEGES.

The professional readers of the COMPANION cannot but be delighted with its enthusiastic efforts in behalf of Medical Education. It is a lamentable fact that the subject is almost eschewed by many of the most prominent journals of the day. And yet it is one which should be held up before the public gaze with unwavering constancy. For all the good to be expected from practical medicine in our time, and in ages to come, is the result of brainwork. The amount of good accomplished will be in exact proportion to the degree of mental cultivation. Experience, without brain-culture, cannot make a man a Doctor. Education guides the practitioner into proper estimates of the facts presented, and enables him to profit by experience. Without it he can never duly appreciate the advantages offered by daily contact with disease. He can never become more than a mere routinist. I am, therefore, more than pleased to see the COMPANION so zealous in the great cause of elevating the standard of medicine through the only possible way—the advance of Medical Education. Your correspondents, many of them, appear to have caught the cue, and are urging their brethren, through the pages of your excellent journal, to come to the rescue, and save science from the dominion of ignorance. The work is a holy one. Let us pursue it with vigor. Let us fight till we conquor the host of pseudo Colleges whose only object is to make money, regardless of consequences—regardless of ethics and integrity—regardless of the ultimate damage to human life consequent upon the multitude of ignorant, stupid and vicious pretenders thrown upon the unsuspecting people—a disgrace to the profession and an incalculable evil to society. Let us lift up the hands of the good and true men battling in and out of Colleges for the benefit of science and a suffering world. If, in your efforts in this direction you can succeed in opening the eyes of our medical brethren throughout the country, your journal should be justly esteemed their truest friend, and should receive the entire endorsement and support of every true professional gentleman.

This comes from one of our most learned and accomplished professional friends. We do indeed hope to see, at no distant day, a grand rally of the profession upon the subject of medical education, and that they, in their power, shall arrest the incalculable evils growing out of the eagerness of inferior medical schools to palm off upon the profession and the public, uneducated doctors. We are rejoiced to know there are many Colleges in every section of this country which have the men and the means of imparting thorough medical educations to students ; and while the profession should patronize these *alone*, they to be consistent and worthy of the confidence of the profession, ought to refuse to recognize inferior and irregular schools in any particular.

